# Antibiotic susceptibility and resistance genes in *Escherichia coli* from broilers reared in a low-antibiotic-use production system

**DOI:** 10.1016/j.psj.2026.106764

**Published:** 2026-03-12

**Authors:** Homayoon Davam, Désirée S. Jansson, Emma Nord, Jesper Rydén, Ingrid Hansson

**Affiliations:** a Department of Animal Biosciences, Swedish University of Agricultural Sciences, SE-750 07 Uppsala, Sweden; b Department of Clinical Sciences, Swedish University of Agricultural Sciences, SE-750 07 Uppsala, Sweden; c Department of Microbiology, Swedish Veterinary Agency, SE-751 89 Uppsala, Sweden; d Department of Energy and Technology, Swedish University of Agricultural Sciences, SE-750 07 Uppsala, Sweden

**Keywords:** Antimicrobial resistance, *Escherichia coli*, Broiler, Resistance gene, Reservoir

## Abstract

Antimicrobial resistance (AMR) is a major global concern for animal and human health. This study investigated the occurrence and patterns of AMR in *Escherichia coli* (*E. coli*) isolated from Swedish broiler flocks reared under low-antibiotic-use conditions. During routine necropsy examinations of 80 broilers from 40 flocks with increased mortality associated with colibacillosis, liver samples were collected for bacteriological analysis. *E. coli* isolated from the liver were classified as clinical *E. coli.* In addition, boot sock samples were taken to collect feces from the litter of 60 broiler flocks with no signs of disease or increased mortality. *E. coli* isolates (*n* = 109) obtained from boot sock samples were classified as non-clinical *E. coli*. Susceptibility to 15 antibiotics was assessed using broth microdilution, and resistance-associated genes and mutations were identified through whole-genome sequencing (WGS). Overall resistance was low, with all isolates susceptible to 9 of the 15 tested antibiotics: meropenem, azithromycin, amikacin, gentamicin, tigecycline, ceftazidime, cefotaxime, chloramphenicol, and colistin. Resistance was significantly more frequent in non-clinical than clinical isolates for the six antibiotics with detected resistance (*P* < 0.05) and was strongly correlated with the presence of known AMR genes or mutations. Among clinical isolates, 93.7% were fully susceptible to all tested antibiotics, compared with 49.5% of non-clinical isolates. The highest resistance rates were observed in non-clinical isolates against ampicillin (34%), sulfamethoxazole (32.1%), and trimethoprim (28.4%). The results of this study indicate that in low-antibiotic-use production systems, factors beyond direct antibiotic use—such as horizontal gene transfer, vertical transmission, and environmental contamination—may contribute to AMR dissemination. Higher AMR rates in non-clinical isolates suggest that these isolates may serve as reservoirs of resistance genes. This highlights the importance of monitoring commensal *E. coli* and farm environments to support AMR mitigation and sustainable broiler production.

## Introduction

*Escherichia coli* (*E. coli*) is a common inhabitant of the intestinal microbiota of most warm-blooded animals, including poultry, typically existing as a commensal (Nolan et al., 2020). However, certain strains, known as avian pathogenic *E. coli* (**APEC**), belong to a subset of extraintestinal pathogenic *E. coli* and carry virulence-associated genes (**VAG**) that enable them to cause various infections in poultry, collectively referred to as colibacillosis. Colibacillosis is one of the most prevalent bacterial infections among poultry, associated with welfare concerns and considerable economic losses (Nolan et al., 2020).

Antimicrobial resistance (**AMR**) poses a significant challenge to human and veterinary medicine. Widespread and sometimes improper antibiotic usage has contributed to the emergence and spread of resistant bacteria in livestock and agricultural environments (Chantziaras et al., 2014). In addition to their therapeutic use, antibiotics are still used as growth promoters for farm animals in numerous countries (Van Boeckel et al., 2015), although this practice has been banned in the EU since 2006 (Regulation (EC) No 1831/2003) and in Sweden since 1986 (Wierup et al., 2021). Regarding Swedish broiler farms, AMR rates are considerably lower compared with most other European countries (European Food Safety Authority (EFSA) and European Centre for Disease Prevention and Control (ECDC), 2024), likely due to restrictive antibiotic use (European Medicines Agency and European Surveillance of Veterinary Antimicrobial Consumption, 2022). Approximately 109 million broilers were produced in Sweden in 2023 (Swedish Board of Agriculture, 2025). In the same year, only 9 out of 3,490 broiler flocks on farms affiliated with the Swedish Poultry Meat Association were treated with antibiotics, all involving the use of phenoxymethylpenicillin for necrotic enteritis, with no treatments reported for colibacillosis (Swedres-Svarm, 2023). According to the Swedish resistance monitoring program, Swedres-Svarm, only 0.4% of Swedish broiler flocks were treated with antibiotics between 2013 and 2023 (Swedres-Svarm, 2023).

In its 2024 update of the Bacterial Priority Pathogens List, the World Health Organization (WHO) classified third-generation cephalosporin-resistant and carbapenem-resistant *Enterobacterales* as critical priority pathogens (WHO, 2024). This classification underscores the urgent need for enhanced surveillance and control measures to combat resistance in *Enterobacterales*, including *E. coli*. Bacteria primarily develop AMR through mutations or horizontal gene transfer (**HGT**), with resistance mechanisms varying based on the antibiotic class and the genetic characteristics of the strain, such as the presence of mobile genetic elements like plasmids, transposons, and integrons (Barlow, 2009). Increased resistance rates to multiple antibiotics have rendered colibacillosis in poultry difficult to treat, with *E. coli* acting as a major reservoir of resistance genes (Bailey et al., 2010). These genes may spread between bacterial populations in animals and humans through direct contact, environmental exposure, the food chain, or via HGT (Marshall and Levy, 2011; Lazarus et al., 2015). Due to these characteristics and their abundance, *E. coli* isolates from the intestinal microbiota of healthy food-producing animals are widely used as indicators in AMR monitoring programs, such as those coordinated by EFSA, to track resistance trends in livestock (EFSA and ECDC, 2024). However, in contrast to indicator *E. coli* isolates, data on AMR in pathogenic isolates remain scarce and lack harmonization across Europe. While some innovative surveillance programs monitor antibiotic susceptibility in pathogenic isolates, available datasets remain limited, and access is often restricted (Mesa-Varona et al., 2020a).

The relationship between AMR and pathogenicity in *E. coli* is complex and not yet fully understood. Although multidrug-resistant (**MDR**) *E. coli* strains are often presumed to be more pathogenic, research findings are inconsistent. Certain studies reported a positive correlation between pathogenicity and AMR, notably when resistance and virulence genes are co-located on the same mobile genetic elements, facilitating co-mobilization under antimicrobial selective pressure (Johnson and Nolan, 2009; Aasmäe et al., 2019). However, other studies observed that highly resistant *E. coli* isolates may exhibit reduced pathogenicity and lower virulence, suggesting a possible trade-off between AMR and pathogenicity (Moreno et al., 2006; Mesa-Varona et al., 2020b). Variability across study findings and limited comparative research on AMR in pathogenic and non-pathogenic *E. coli* from poultry necessitate further investigation. Advancements in whole-genome sequencing (**WGS**) have considerably enhanced our ability to characterize resistance. In Swedish poultry production, minimal antibiotic use offers a unique opportunity to study AMR mechanisms in the relative absence of antibiotic selective pressure.

This study aimed to assess AMR in *E. coli* isolated from Swedish broiler flocks by analyzing the distribution of resistant isolates from flocks with increased mortality due to colibacillosis (clinical isolates) and comparing them with isolates from flocks with normal mortality rates and no clinical signs of disease (non-clinical isolates). Furthermore, the objective was to determine the genetic basis of resistance by identifying resistance genes and mutations using WGS.

## Materials and methods

### *Study Population*

Samples were collected from conventional broiler farms in Sweden. All farms were affiliated with the Swedish Poultry Meat Association, which represents approximately 98% of Swedish broiler production. In Sweden, litter is never reused in broiler production, and barns are cleaned and disinfected between production cycles. All broilers were of the Ross 308 strain. The sampling period extended from November 2022 to May 2024, and samples were collected from broilers of varying ages, ranging from 4 to 35 days.

### *Classification of Flocks and Sampling Procedures*

A total of 189 *E. coli* isolates were collected. Of these, 80 were obtained from the livers of necropsied broilers from 40 flocks (two per flock) across 26 farms experiencing increased mortality due to colibacillosis (referred to as “clinical isolates”). Additionally, 109 isolates (one to two per flock) were collected from the litter using boot sock sampling in 60 flocks without increased mortality or signs of disease on 26 farms (referred to as “non-clinical isolates”). Boot sock sampling was used to collect feces in order to avoid invasive sampling and minimize animal handling and welfare impacts. This method has been repeatedly shown to be a reliable proxy for fecal sampling and intestinal contents in chickens and has been successfully used for monitoring AMR in *E. coli* (Bortolaia et al., 2010), detection of *Campylobacter* spp. (Frosth et al., 2020; Joensen et al., 2025), characterization of the broiler intestinal microbiota (Kers et al., 2019), and *Salmonella* spp. prevalence screening in broilers within the EU (Regulation (EC) No 646/2007). Multiple flocks from the same farm could be included during a single production cycle; however, only one sample per flock was collected within each cycle. Thirteen farms contributed samples to both the clinical and non-clinical categories, but in such cases, samples were obtained from different flocks and, except in one case, from separate production cycles.

An isolate was classified as clinical if it originated from a flock with a sudden increase in mortality suspected to be caused by colibacillosis, together with post-mortem findings indicative of the disease. Specifically, gross lesions consistent with colibacillosis had to be observed in more than 50% of the chickens submitted for necropsy. From each flock, 5–10 chickens, either recently dead or euthanized for animal welfare reasons, were transported overnight to the Swedish University of Agricultural Sciences (SLU) or the Swedish Veterinary Agency (SVA) for necropsy and sampling. All necropsies were performed by the same veterinarian following a standardized in-house necropsy protocol based on the guidelines described by Brugère-Picoux et al. (2015). Broilers with gross lesions consistent with colibacillosis and low-grade autolysis were selected for sampling. Samples were collected from the liver parenchyma using sterile swabs (Copan Diagnostics, Inc., Murrieta, CA, USA) following surface searing.

Isolates were classified as non-clinical if they were obtained from flocks without increased mortality, as reported by farmers and veterinarians, for at least seven days prior to sampling and with no clinical signs of disease. Samples from these flocks were collected using boot sock samples (Danafast 7.5, Tubular Retention Bandage, Mediplast AB, Malmö, Sweden), following the method described by Hansson et al. (2020). The socks were moistened with 30 mL of Buffered Peptone Water (BPW) (Oxoid, Basingstoke, UK) and worn over shoes before walking on the litter. The sock samples were then placed in plastic stomacher bags and shipped at ambient temperature to SLU for bacteriological analyses.

### *Bacteriological analyses*

Swabs collected from the liver were cultured directly on 5% bovine blood agar (BA) plates (SVA, Uppsala, Sweden), following standardized methods, and incubated at 37 ± 1°C for 18–24 hours. Boot sock samples were enriched by adding 90 mL BPW in stomacher bags, followed by homogenization in a stomacher for 1 minute at 240 rpm. After enrichment at 37 ± 1°C for 18–24 hours, two loopfuls (approximately 20 µL) were cultured on MacConkey agar (MAC) plates (SVA, Uppsala, Sweden) and incubated at 37 ± 1°C for 18–24 hours. Two presumed *E. coli* colonies were selected per plate based on colonial morphology and identified using MALDI-TOF MS (Bruker Daltonics, Germany). Confirmed *E. coli* colonies were re-cultured on BA plates to ensure purity and incubated for 18–24 hours at 37 ± 1°C. All isolates were stored at −70°C in brain–heart infusion (BHI) broth (CM1135; Oxoid) with 15% glycerol.

### *Antibiotic susceptibility testing*

Susceptibility to 15 selected antibiotics (Table 1) was assessed by determining the minimum inhibitory concentration (**MIC**) using the broth microdilution method with Sensititre EU Surveillance *Salmonella/E. coli* EUVSEC3 AST Plates (Thermo Fisher Scientific, Waltham, MA, USA), following the manufacturer’s instructions for inoculum preparation and incubation. Quality control was performed using *E. coli* reference strain ATCC 25922. MIC values were interpreted using the European Committee on Antimicrobial Susceptibility Testing (EUCAST) epidemiological cut-off (ECOFF) values (EUCAST, 2025). The ECOFF values distinguish isolates without phenotypically detectable acquired resistance mechanisms (wild type) from those with such mechanisms (non-wild type). In this study, non-wild type isolates are referred to as ‘resistant’, consistent with the Swedish resistance monitoring program (Swedres-Svarm, 2023). However, it should be noted that this classification is intended for surveillance purposes and does not necessarily indicate clinical resistance. Multidrug resistance was defined as resistance to three or more antibiotic classes. Ciprofloxacin and nalidixic acid were grouped under the same antibiotic class (quinolones), while sulfamethoxazole and trimethoprim were classified into separate classes due to their different mechanisms of action. Isolates were suspected to be extended-spectrum β-lactamase (**ESBL**)-producing if they exhibited reduced susceptibility to cefotaxime (MIC > 0.25 mg/L) and/or ceftazidime (MIC > 1 mg/L).

**Table 1:** Distribution of minimum inhibitory concentration (MIC) (mg/L) in non-clinical (*n* = 109) and clinical (*n* = 80) *Escherichia coli* isolates from broilers in Sweden. Results are presented as the percentage of isolates at each MIC. White fields indicate the tested dilution range, and bold vertical lines show the cut-off values used to define resistance. MIC values below or above the tested concentration range are reported as the lowest or highest concentration, respectively.

### *Whole-genome sequencing*

DNA extraction was conducted using magnetic-particle technology with the EZ1 Advanced XL instrument and EZ1 DNA Tissue Kit (Qiagen, Hilden, Germany), following the manufacturer’s instructions. The DNA concentration was measured using the Qubit dsDNA High Sensitivity Assay Kit on a Qubit 2.0 Fluorometer (Invitrogen, Carlsbad, CA, USA). Library preparation was performed using the Nextera XT DNA Library Preparation Kit (Illumina, San Diego, CA, USA), and library quality was assessed with the High Sensitivity DNA ScreenTape D1000 on the 4150 TapeStation System (Agilent Technologies, Santa Clara, CA, USA). Prepared DNA libraries were sent to SciLifeLab Clinical Genomics (Solna, Stockholm) for sequencing on an Illumina NovaSeq X instrument, generating 2 × 150 bp reads. Reads were quality-checked and trimmed using fastp v. 0.23.4 (Chen, 2023), downsampled to approximately 100× coverage with BBMap reformat v. 39.09 (Bushnell, 2025), and *de novo* assembled with Unicycler v. 0.5.1 (Wick et al., 2017). Trimmed reads were checked for contamination with Kraken2 v. 2.1.3 (Wood et al., 2019), and assembly quality was assessed using QUAST v. 5.0.2 (Mikheenko et al., 2018). Resistance genes and resistance-associated mutations were identified with ResFinder v. 4.6.0 (Camacho et al., 2009; Bortolaia et al., 2020) using the ResFinder database v. 2.4.0 and PointFinder database v. 4.1.1. Thresholds of 90% sequence identity and 90% minimum length coverage were applied for the identification of acquired resistance genes and point mutations. All raw sequencing data for this article have been submitted to the European Nucleotide Archive under accession number PRJEB89812.

### *Statistical analysis*

Differences in resistance between clinical and non-clinical isolates were evaluated using logistic regression models, with farm included as a random intercept to account for clustering of isolates within farms. Models were initially fitted with a standard binomial family in R (R Core Team, 2025). Model diagnostics indicated underdispersion (residual deviance markedly smaller than residual degrees of freedom), so models were refitted using a quasibinomial family, which adjusts standard errors while leaving coefficient estimates unchanged (McCullagh, 1989). Exact 95% confidence intervals (CIs) for proportions were calculated using the Clopper–Pearson method (Clopper and Pearson, 1934), computed in R with the *binom* package. A *P* value < 0.05 was considered statistically significant.

### *Ethical approval and farmers' consent*

Animal ethics approval was not required since only dead or culled chickens were subjected to necropsy, i.e., no chickens were euthanized for research purposes, and no invasive sampling was performed. All participating broiler farmers provided informed consent after receiving information about the study’s purpose, data use, and confidentiality.

## Results

### *Antibiotic susceptibility*

All 189 *E. coli* isolates were susceptible to 9 of the 15 tested antibiotics: meropenem, azithromycin, amikacin, gentamicin, tigecycline, ceftazidime, cefotaxime, chloramphenicol, and colistin (Table 1). Resistance rates were significantly higher in non-clinical isolates (*P* < 0.05) for the six antibiotics with detected resistance: ampicillin, tetracycline, sulfamethoxazole, trimethoprim, ciprofloxacin, and nalidixic acid (Table 2). Farm-level clustering analyses indicated a significant farm effect across all six antibiotics (*P* < 0.001).

**Table 2:** Proportion of resistance (%) with 95% confidence intervals (CI) for antibiotics with detected resistance among non-clinical (*n* = 109) and clinical (*n* = 80) *Escherichia coli* isolates from broilers in Sweden.

Antibiotic	Non-clinical isolates, % resistant (95% CI)	Clinical isolates, % resistant (95% CI)	*P* value^a^
Ampicillin	34.0 (25.1–43.6)	3.7 (0.8–10.6)	< 0.001
Sulfamethoxazole	32.1 (23.5–41.7)	0.0 (0–4.5)	< 0.001
Trimethoprim	28.4 (20.2–37.9)	0.0 (0–4.5)	< 0.001
Tetracycline	18.3 (11.6–26.9)	2.5 (0.3–8.7)	< 0.001
Ciprofloxacin	7.3 (3.2–14.0)	0.0 (0–4.5)	0.011
Nalidixic acid	6.4 (2.6–12.8)	0.0 (0–4.5)	0.015

a *P* value < 0.05 was considered statistically significant

Overall, 93.7% (95% CI: 86.0–97.4) of clinical isolates and 49.5% (95% CI: 39.8–59.3) of non-clinical isolates were fully susceptible to all tested antibiotics. Only 5 of the 80 clinical isolates (6.3%) showed resistance, each to a single antibiotic, either ampicillin or tetracycline. In contrast, among non-clinical isolates, 13.7% (15/109) were resistant to one antibiotic, 9.2% (10/109) to two, 17.4% (19/109) to three, 9.1% (10/109) to four, and 0.9% (1/109) to five. Multidrug resistance was observed exclusively in non-clinical isolates, with 27.5% (30/109) resistant to three or more antibiotic classes. None of the *E. coli* isolates were phenotypically identified as ESBL-producing.

### *Resistance genes and mutations*

All resistance genes and mutations, except in one case, aligned with phenotypic resistance profiles for their target antibiotics. The exception was an isolate that exhibited phenotypic sulfamethoxazole resistance despite lacking any known sulfonamide resistance gene (Table 3). A mutation in *gyrA* with the S83L amino acid substitution was identified in all isolates resistant to both ciprofloxacin and nalidixic acid. The single isolate resistant to ciprofloxacin but susceptible to nalidixic acid carried the *qnrS1* gene. Although mutations in *parC* and *parE* were also detected, isolates with these mutations alone were not phenotypically resistant to quinolones. The mutation in *parE* was observed exclusively in clinical isolates and was the only mutation more frequently found in clinical than non-clinical isolates. Genes encoding aminoglycoside-modifying enzymes, including *aadA1* and various *aph* variants, were also detected and were more frequent in non-clinical isolates. However, because their target antibiotics were not included in the susceptibility testing plate, phenotypic resistance associated with these genes could not be assessed.

**Table 3:** Detected resistance genes and mutations in non-clinical (*n* = 109) and clinical (*n* = 80) *Escherichia coli* isolates from broilers in Sweden.

Gene or mutation^a^	Target antibiotic(s)	No. of non-clinical isolates with gene/mutation detected	No. of clinical isolates with gene/mutation detected
*bla_TEM_*_-1B_	Ampicillin	37	2
*bla_TEM_*_-1C_	Ampicillin	0	1
*dfrA1*	Trimethoprim	30	0
*dfrA5*	Trimethoprim	1	0
*tet*(A)	Tetracycline	20	2
*sul2*	Sulfamethoxazole	34	0
*aadA1*	Spectinomycin, streptomycin	13	0
*aph(3′)-Ia*	Neomycin, kanamycin, lividomycin, paromomycin, ribostamycin	1	0
*aph(3′')-Ib*	Streptomycin	8	1
*aph(6)-Id*	Streptomycin	8	1
*qnrS1*	Ciprofloxacin	1	0
*gyrA* (p.S83L)	Nalidixic acid, ciprofloxacin	7	0
*parC* (p.S57T)	Nalidixic acid, ciprofloxacin	2	0
*parE* (p.I355T)	Nalidixic acid, ciprofloxacin	0	10

a Gene names are shown in italics; mutations are indicated by amino acid substitutions

## Discussion

This study highlights notable differences in AMR between clinical and non-clinical *E. coli* isolates from broiler farms in Sweden. Isolates were classified based on whether they originated from flocks with or without elevated mortality rates and diagnosed colibacillosis, rather than on the presence of VAGs. The reliability of VAGs as APEC markers is debated, as they may also be present in presumed commensal *E. coli* residing in the intestines of healthy birds (Collingwood et al., 2014). To avoid assumptions about virulence potential, we used "clinical" and "non-clinical" rather than “APEC” and “commensal.”

Overall, AMR rates in *E. coli* were low compared with surveillance data from most European countries (EFSA and ECDC, 2024), likely reflecting the effectiveness of Sweden's strict antibiotic use policies (Wierup et al., 2021). Moreover, no ESBL-producing *E. coli* were detected, consistent with national efforts to eliminate such isolates from broiler production (Nilsson et al., 2020). In 2010, a high proportion of Swedish broilers were colonized with ESBL-producing *E. coli*, as determined by antibiotic susceptibility testing, and were likely introduced through imported broiler breeders (grandparent generation). In response, a screening program for imported day-old chickens was implemented, which helped reduce the prevalence of ESBL-producing isolates in imported chicks from 35% in 2015 to zero by 2018 (Nilsson et al., 2020). As a result, the prevalence of ESBL-producing *E. coli* isolates in Swedish broilers significantly decreased, from around 40% in 2015 and 2016 to below 5% between 2019 and 2023 (Swedres-Svarm, 2023).

Despite extremely low antibiotic usage in Swedish broiler production, notable resistance to several antibiotic classes was observed in non-clinical isolates, suggesting that selective pressure from antibiotic use alone cannot explain the observed resistance patterns. Our models also identified a significant farm-level effect for all six antibiotics with detected resistance (*P* < 0.001), indicating uneven distribution across farms and underscoring the role of farm-level factors in shaping AMR patterns. Beyond direct antibiotic use, several mechanisms may contribute to the emergence and persistence of AMR in *E. coli* within broiler production. These include mechanisms of gene transfer (such as HGT), vertical transmission, and environmental reservoirs of resistant bacteria. Horizontal gene transfer enables bacteria to acquire or transfer resistance genes via mobile genetic elements, particularly during co-infection or within the intestinal microbiota, where close bacterial interactions facilitate gene exchange (Aminov and Mackie, 2007; Poirel et al., 2018). The frequent occurrence of mutations and HGT in *E. coli* contributes to its genomic plasticity and underlines its capacity to rapidly acquire AMR (Rasko et al., 2008; Mageiros et al., 2021). Vertical transmission within the breeding pyramid may also influence AMR dissemination, as resistant *E. coli* in the gastrointestinal or reproductive tract of breeder chickens can be transmitted to their offspring, a pathway that can persist despite strict hygiene and biosecurity measures (Bortolaia et al., 2010; Poulsen et al., 2017). Additionally, environmental contamination may contribute to AMR persistence. Even in the absence of antibiotic use, the farm environment—both inside the broiler house and in surrounding areas—can act as a reservoir where resistant bacteria persist and recirculate (Allen et al., 2010; Leclercq et al., 2024), even after cleaning and disinfection between consecutive flocks (Dierikx et al., 2013). In our study, the highest resistance rates were observed for ampicillin, sulfamethoxazole, trimethoprim, and tetracycline. These antibiotics are among the most sold for veterinary use across animal species in Sweden throughout the past decade (Swedres-Svarm, 2023), suggesting that environmental contamination may have influenced the development of resistance in non-clinical isolates. Notably, non-clinical isolates from the litter may include *E. coli* from sources other than the intestinal microbiota of broilers, which should be considered when interpreting resistance findings in this group.

Resistance rates among indicator *E. coli* isolated from broilers vary across European countries. According to EFSA monitoring, the highest proportions of fully susceptible indicator *E. coli* in 2022 were reported in Nordic countries, with Sweden at 68.7%, Norway at 79.3%, and Finland and Iceland both at 80% (EFSA and ECDC, 2024), all of which apply a restrictive antibiotic use policy. In contrast, countries with more intensive antibiotic usage reported lower full susceptibility rates, such as Germany (17.9%), Spain (21.8%), and France (43.4%) (EFSA and ECDC, 2024). The full susceptibility rate among non-clinical isolates in this study (49.5%) was lower than that reported by EFSA, possibly due to differences in study design or flock inclusion criteria. Nonetheless, the overall full susceptibility rate across all *E. coli* isolates in this study (68.2%) closely aligns with EFSA’s findings.

Inconsistent reporting of AMR data for *E. coli* isolated from broilers with colibacillosis renders direct comparisons challenging. Differences in sampling strategies and methodologies—including laboratory techniques, applied standards (e.g., EUCAST vs. CLSI), interpretation criteria, and antimicrobial panels—further complicate cross-study comparisons. In a German study, only 16.2% of isolates from broilers with colibacillosis were fully susceptible, with the highest resistance to ampicillin (53%), streptomycin (47%), and nalidixic acid (34%) (Müller et al., 2024). In Norway, AMR rates among *E. coli* isolated from broilers with colibacillosis were substantially lower, with resistance to ampicillin and gentamicin at 2.3%, and to nalidixic acid and tetracycline at 4.7% in 2016, increasing to 10.4%, 3.9%, 13%, and 16.9%, respectively in 2017 (Mesa-Varona et al., 2021). In our study, 93.7% of the clinical isolates were susceptible to all antibiotics, with only five isolates showing resistance to a single antibiotic. Outside Europe, AMR rates in *E. coli* from broilers are generally higher. In Brazil, there was no antibiotic to which all the *E. coli* isolates were susceptible (Barbieri et al., 2015), and in Egypt, all isolates were MDR (Radwan et al., 2020). Moreover, full susceptibility rates among *E. coli* from broilers with colibacillosis were 34.4% in the USA (Newman et al., 2021), and 15.7% in Canada (Varga et al., 2018).

Our findings demonstrated significantly higher resistance rates in non-clinical compared with clinical isolates (*P* < 0.05). Studies directly comparing AMR in *E. coli* from flocks with and without clinical signs of disease under identical study conditions are limited. Mesa-Varona et al. (2021) compared AMR of *E. coli* from broilers with and without signs of colibacillosis reared in Germany, France, Norway, and the UK, and reported a general trend of higher AMR in *E. coli* from broilers without signs of disease. Among the tested antibiotics with significant differences in resistance rates, ampicillin and tetracycline resistance were lower in *E. coli* isolates from diseased broilers from France, Germany, and the UK, while gentamicin resistance was higher in *E. coli* isolates from diseased broilers from France (Mesa-Varona et al., 2021). In settings with generally higher AMR levels, detectable differences between *E. coli* from flocks with and without signs of colibacillosis may be reduced. In Qatar, *E. coli* isolates from healthy and diseased broilers within the same flock displayed a 99.3% MDR rate (Johar et al., 2021). However, *E. coli* from broilers with colibacillosis exhibited significantly higher resistance to ampicillin, chloramphenicol, and colistin, whereas *E. coli* isolates from broilers not showing any signs of disease were more resistant to trimethoprim-sulfamethoxazole (Johar et al., 2021). Similarly, a Brazilian study found only 11.1% full susceptibility in both APEC and less virulent avian fecal *E. coli* (**AFEC**) isolates from broilers. Although differences were not statistically significant, MDR was more frequent in AFEC (53%) than APEC (29.4%) (Lúcio et al., 2025).

The strong correlation between phenotypic resistance and the presence of known AMR genes or mutations among our isolates supports the use of molecular screening as a reliable tool for AMR detection. All isolates resistant to ampicillin, trimethoprim, and tetracycline carried *bla*_TEM-1_, *dfrA* and *tet*(A)*,* respectively. *Bla*_TEM-1_, typically plasmid-borne, mediates β-lactam resistance through enzymatic inactivation (Bradford, 2001). *DfrA*, commonly located in class 1 or 2 integrons, confers trimethoprim resistance, while *tet*(A) encodes a tetracycline efflux pump and is frequently found on both conjugative and non-conjugative plasmids (Poirel et al., 2018). All but one sulfamethoxazole-resistant isolate carried *sul2*, a plasmid-associated gene often found in animal-derived *E. coli* and frequently co-located with other AMR genes (Poirel et al., 2018). The remaining resistant isolate lacked any *sul* gene, suggesting an alternative resistance mechanism. Resistance to both ciprofloxacin and nalidixic acid was consistently associated with a mutation in *gyrA* with the S83L amino acid substitution, a well-documented driver of quinolone resistance (Redgrave et al., 2014). Isolates with mutations in *parC* and *parE* in the absence of a *gyrA* mutation remained phenotypically susceptible, consistent with findings that the impact of *parC* or *parE* mutations in *E. coli* often depends on the presence of a concurrent *gyrA* mutation (Collins and Osheroff, 2024).

The resistance genes identified in this study have commonly been reported on mobile genetic elements in *E. coli*. Resistance genes situated on the same mobile genetic element can be co-transferred, facilitating co-selection (Alonso et al., 2017; Dominguez et al., 2018), which may contribute to the higher occurrence of resistance genes in non-clinical isolates despite minimal antibiotic use. All MDR isolates in our study originated from the non-clinical group, and 96.6% (29/30) co-harbored *bla*_TEM-1_, *dfrA1* and *sul2*, accounting for 75% of ampicillin-resistant, 96.7% of trimethoprim-resistant, and 85.7% of sulfamethoxazole-resistant isolates. The high genetic diversity of intestinal and environmental *E. coli* suggests a strong potential for HGT, and a stable intestinal microbiota may further facilitate the persistence and dissemination of resistance genes (Djordjevic et al., 2013; Card et al., 2017). This contrasts with the near absence of resistance in clinical isolates, suggesting that in low-antibiotic-use broiler production systems, such as in Sweden, successful pathogenic strains may rely more on virulence factors than on acquired resistance genes for pathogenicity. Studies have shown that carriage of some AMR genes can reduce bacterial fitness in the absence of antibiotic pressure (Rajer and Sandegren, 2022), and certain resistance-associated mutations, such as those in *gyrA*, can reduce the expression of several virulence factors and pathogenicity in *E. coli* (Sánchez-Céspedes et al., 2015). Consistent with this, Ronco et al. (2017) identified a clonal APEC lineage dominating colibacillosis cases in Nordic broilers that harbored a broad and conserved repertoire of virulence genes but contained fewer resistance genes than other, less common lineages.

In conclusion, this study provides a baseline for understanding resistance patterns from broilers reared in a low-antibiotic-use production system. The higher occurrence of resistance among *E. coli* from broiler flocks without clinical signs of disease raises concerns that intestinal and environmental isolates may represent important reservoirs of resistance genes, with the potential for these genes to be transferred to pathogenic strains. Future studies should prioritize investigating the genetic mechanisms underlying resistance, with particular attention to the relative roles of vertical transmission and HGT in disseminating resistance within poultry production systems. Such efforts are essential for developing targeted interventions to safeguard both animal and public health.

## Funding

This study was funded by the Swedish Farmers’ Foundation for Agricultural Research [grant agreement number O-21-20-619]. The funder played no role in study design, data collection, analysis, and interpretation of data, or the writing of this manuscript.

## Disclosures

The authors declare that they have no known competing financial interests or personal relationships that could have appeared to influence the work reported in this paper.
